# Interactive Narrative in a Mobile Health Behavioral Intervention (Tumaini): Theoretical Grounding and Structure of a Smartphone Game to Prevent HIV Among Young Africans

**DOI:** 10.2196/13037

**Published:** 2019-05-08

**Authors:** Kate Winskell, Gaëlle Sabben, Christopher Obong'o

**Affiliations:** 1 Hubert Department of Global Health Rollins School of Public Health Emory University Atlanta, GA United States; 2 School of Public Health University of Memphis Memphis, TN United States

**Keywords:** mHealth, serious games, games for health, narrative, HIV, adolescence, sub-Saharan Africa, behavioral theory, narrative theory

## Abstract

The increasing availability of smartphones, including in low-income countries, offers an unprecedented opportunity to reach individuals with innovative health promotion interventions. Electronic games delivered via smartphone offer promising avenues for sexual health promotion and HIV prevention, especially for young people. By giving players real agency in a virtual and safe environment, well-designed games can provide a level of experiential learning unparalleled by many other behavioral interventions. The design of effective games for health relies on multidisciplinary insight and expertise. However, relatively few studies discuss the theoretical understanding underlying their intervention. Making explicit the theoretical grounding of a game-based intervention allows articulation of assumptions and strategies, anticipation of outcomes, and evaluation of impacts (including intermediate effects), thereby increasing understanding of pathways to change, with a view to contributing to the development of more effective games. It also helps strengthen the credibility and improve the accountability of games for health. We present the multidisciplinary theoretical framework—integrating intervention design, mediators, and behavioral outcomes—and the structure of an HIV prevention game for young African adolescents that has shown promise in a randomized pilot study in Western Kenya. The central component of *Tumaini* (*hope for the future* in Kiswahili) is an interactive role-playing narrative in which the player makes choices for characters that determine how their paths unfold. In addition, a series of mini-games reinforce skills, and the “My Story” component links the game world to the player’s own life and goals, and a reward system motivates continued play. With its “choose-your-own-adventure” format, *Tumaini* is intended to be replayed so that players can experience the consequences resulting from different choices made in the role-playing narrative. Grounded in theories of narrative and applied communication and in social behavioral theories, especially Social Cognitive Theory, *Tumaini* is designed to help young adolescents acquire the information, skills, and motivation they need to avoid and reduce sexual risks. We close by situating *Tumaini* within discussion of the theory and practice of using interactive narrative in health promotion, with a view to furthering theoretical elaboration.

## Introduction

A third of all new adult HIV worldwide infections occur in young people aged between 15 and 24 years [1]. Although young women are particularly vulnerable because of biological and structural factors, both sexes too often lack the understanding, skills, and guidance they need to successfully navigate the physiological, emotional, social, and behavioral changes and challenges of adolescence and avoid or reduce risks to their sexual health. They need help in identifying new risk situations to which they will be exposed and in which gendered pressures—structural, normative, group, and interpersonal—constrain their ability to make healthy choices. Prominent cultural scripts may leave them feeling little control over sexual situations [2,3], and they may need to learn how to exercise agency. Ideally, they need opportunities to prepare for potential risk situations through interventions informed by behavioral theory that allow them to build and practice skills.

Although role-play has been a staple of evidence-based group HIV prevention interventions, in-person exercises have limitations when it comes to successfully simulating real-life contextualized experience [4]. They are dependent on the capacities of the facilitator and the imaginative faculties of participants and may be perceived as embarrassing or inauthentic. A growing number of group interventions for HIV prevention among adolescents have shown efficacy in a randomized controlled trial (RCT) in a high-resource country. However, such interventions may face adaptation and implementation challenges, including the high cost of facilitator-led group sessions and their poor record of translation from RCT to community setting [3]; these challenges may be magnified in low-resource settings.

Computer-based (electronic health or “eHealth”) interventions for HIV prevention have shown positive effects on behavioral mediators and clinical outcomes [5,6]. A new generation of interventions on mobile devices often incorporate higher levels of interactivity and personalization, recognized as increasing effects on behavior. Increasingly accessible smartphone technologies make it possible to engage youth—at scale and at low cost—in culturally adapted prevention interventions that require little personnel to implement with consistent quality, have high entertainment and motivation appeal, and incorporate automated data collection. These prevention interventions can be integrated into everyday life and have the potential to allow young people to learn about sensitive issues in a private virtual space and to determine the pace of their own learning, ideally in line with their developmental needs. A systematic review has shown the potential of interventions using new digital media to improve adolescent sexual and reproductive health [7]. There is recognition of the need for future research to build a stronger evidence base by tracking behavioral outcomes, rather than mediators, over longer periods [7,8]. There is also a need to build an evidence base for such interventions in low- and middle-income countries, where limited research has been conducted to date.

Digital games represent a promising intervention strategy, especially for youth HIV prevention [9]. By allowing players, through interactivity, to experience real agency in a virtual and safe environment, well-designed games provide a level of experiential learning unparalleled by many other interventions. A small but growing body of evidence supports the effectiveness of games for health outcomes [10-13]. In their 2015 systematic review and meta-analysis of games for sexual health, DeSmet et al [11] found positive effects, albeit of small size, on behavioral determinants. They concluded that greater reliance on immersive and health-promoting features that draw on behavioral change and educational gaming literature (specifically role-playing, simulation, and narrative) is likely to increase the effectiveness of the next generation of games for sexual health.

Several studies have questioned the adequacy of current behavioral theories to account for the distinctive affordances of health promotion using new digital media. It has been suggested that there is a need to develop new theories that acknowledge the multidisciplinary background and genealogy of such interventions [7,14-16]. However, few interventions—including games for health—are explicit about their theoretical foundations [8], thereby limiting the potential to elaborate new models and increase the understanding of mechanisms of effect, essential for the development of more effective intervention approaches [17]. Where theory is addressed, it focuses on psychosocial constructs from behavioral science theory and rarely incorporates multidisciplinary perspectives [16].

We present here the theoretical grounding and structure of a smartphone game for HIV prevention using interactive narrative that has shown promise in a pilot randomized study [18]. The game is designed to provide African preadolescents (aged 11-14 years) with the knowledge, motivation, and behavioral skills to delay sexual initiation and use a condom from first sex. *Tumaini* (*hope for the future* in Kiswahili) is a game-based intervention built around an extensive interactive narrative or *choose-your-own-adventure* (CYOA) format. It was created with a US commercial game developer and with input from US-based and Kenyan specialists in adolescent sexual and reproductive health and from Kenyan preadolescents and their parents. *Tumaini* is grounded in distinctive research on HIV-related narratives written by young people aged 10-24 years across sub-Saharan Africa. The narratives, submitted by tens of thousands of young Africans to Global Dialogues/Scenarios from Africa [19] scriptwriting contests, provide age-specific insight into youth sexual culture, decision-making, and sociocultural context [20-27]*.*

In a randomized controlled pilot study in Kisumu, Western Kenya, with 60 participants aged 11-14 years, intervention arm participants (n=30) played *Tumaini* over 50% longer than instructed, a mean of approximately 27 hours over a 16-day period [18]. Control arm participants received no intervention beyond any existing sex education from family, school, and peers [28]. The intervention arm showed significant gains in sexual health-related knowledge and self-efficacy (both *P*<.001), behavioral intention for risk-avoidance strategies and sexual risk communication (*P*=.006), and overall survey scores (*P*<.001) compared with the control arm 6 weeks postintervention. A postintervention survey revealed high subjective measures of the game’s value, relevance, and appeal, and postintervention focus groups with preadolescents and their parents identified a wide range of knowledge and skills they had gained. We present the theoretical grounding and structure of *Tumaini* here, with a view to furthering theoretical elaboration of both games for sexual health and games rooted in interactive narrative.

## Theoretical Framework

**Figure 1:**
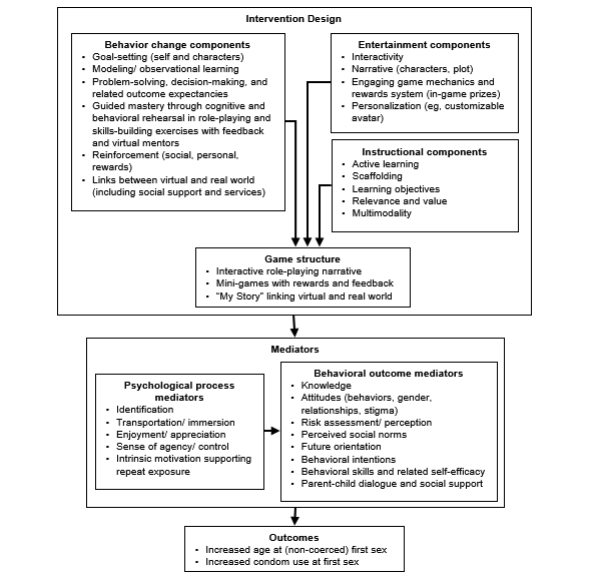
Theoretical framework for Tumaini, an HIV prevention game rooted in interactive narrative.

*Tumaini* is grounded in (1) theory on narrative and narrative-based applied communication, (2) social behavioral theory and existing evidence-based HIV prevention interventions, and (3) principles of instructional design. Following Thompson et al [29], the theoretical framework is divided into 3 parts: intervention design, mediators, and outcomes. In our theoretical framework (Figure 1), these are represented by (1) the entertainment components of intervention design and the psychological process mediators, (2) the behavior change components of intervention design and behavioral outcome mediators, and (3) the instructional components applied in intervention design. Although we separate the 3 sets of mediators for clarity and ease of measurement, they are interlinked and are integrated within the game structure and our overarching theory of interactive narrative and its effects.

*Tumaini* is made up of 3 integrated parts (Multimedia Appendix 1): (1) a central interactive narrative featuring 6 playable characters (3 male and 3 female characters) whom players guide into and through adolescence, making decisions that have short- and long-term consequences for the characters’ lives, relationships, and health; (2) a set of mini-games that tie into the narrative and support and reinforce its core themes; and (3) “My Story,” in which the player sets goals, plans how to achieve them, and reflects on how the game relates to his/her life and how he/she might apply the lessons learned to protect his/her future. To respect the integration of theory and design in our narrative-based intervention, we contextualize the mediators identified in our theoretical framework (which we subsequently italicize for ease of identification) within theory on narrative and narrative-based applied communication and within behavioral theories first, before describing its structure in greater depth.

### Narrative: Entertainment Components and Psychological Processing Mediators

New digital media provide platforms for unprecedented forms of interactive learning, facilitating learners’ ownership of knowledge and skills and their application in real life. *Tumaini* ’s CYOA structure combines *interactivity* with *narrative*, while incorporating *engaging game mechanics and rewards system*, and *personalization*, which are common features of games for health.

#### Narrative Processing

The past two decades have seen increasing interest in the use of narrative in social and behavior change communication [30], including games for health. There is growing recognition among practitioners and applied communication specialists of the opportunities narrative offers to contextualize learning within relevant, meaningful, and emotionally resonant situations and storylines, thereby increasing both audiences’ motivation to engage with the subject matter and the potential for transferal of learning to real life. Communication scholars argue that narrative is easier to remember than non-narrative communication and also easier to understand and therefore more accessible [31]. It has the advantage of being closer than non-narrative communication to lived experience in its simultaneous appeal to multiple senses, to reason and emotion, to intellect and imagination, and to fact and value. Indeed, in narratives, we are able to “perfink,” or perceive, feel, and think simultaneously [32].

Narratives have the potential to generate in us real and powerful emotions [33,34]: these allow us to live, experience, and learn vicariously, obviating the need to learn through direct personal experience. Through the embodied emotion that narratives generate, in large part through our *identification* with characters, the story experience becomes a personal experience. *Transportation* —whereby individuals suspend disbelief and are carried away imaginatively into the narrative’s world—distinguishes the processing of narrative from other forms of communication [35]. Related to *immersion* in the game literature, transportation is believed to be a primary driver of the persuasive appeal of narratives, that is, the fact that narratives are more difficult to resist, counterargue, or refute than non-narrative communication and therefore have a greater impact on attitudes [36].

Transportation is recognized as an enjoyable experience; however, some scholars differentiate between *enjoyment*, a form of hedonistic pleasure, and *appreciation*, a form that is valued for being meaningful, moving, and/or thought-provoking [37]. Because they are pleasurable, narratives can provide a “self-motivating vehicle for information delivery” [38]. They also facilitate sense-making and recall, by providing a framework for organizing new knowledge that builds on existing knowledge or familiar situations. Narratives are particularly well-suited for conveying complex phenomena and processes, in part because of their ability to accommodate multiple voices and perspectives; this can make even complex processes seem “more accessible, intuitive and memorable” [38]. In addition, by contextualizing information, they can “create memory traces that automatically bring strategies to mind when those future contexts arise” [38].

#### Interactive Narrative

Beyond the emotional and cognitive participatory responses that narrative evokes (eg, by encouraging us to engage in anticipatory problem-solving) [39], some narratives—for example, CYOA novels—are deliberately constructed to be interactive. In contrast to a traditional narrative in which the author has complete control over the characters and plot progression, interactive narrative is “a story in which the reader has the opportunities to decide the direction of the narrative, often at a key plot point” [40]. In these narratives, the player assumes the dual role of audience for and coauthor of the story, shifting the power dynamic between audience and author [41].

Narrative-based public health interventions can foster *agency* on the part of the audience in a variety of ways, with a view to promoting critical thinking, problem-solving, and decision-making [30]. Within legacy media, role-play has traditionally assumed a central place in behavioral skills–building training. A striking example is Forum Theater, based on Augusto Boal’s Theater of the Oppressed [42], in which the “fourth wall” is dissolved at a certain point in the action and audience members are invited to come on stage as “spectactors” and try out their own skills at resolving the conflict.

A recent account of the processes and outcomes of interactive narratives focuses on text-based stories, specifically CYOA books [40]. New communication technologies take the potential for interactive engagement with narrative to a new level by offering efficient and convenient interfaces and allowing audiences to assume an increasingly active role, influencing characters and events.

The empirical evidence about the processes and outcomes of interactive narratives is distributed across a range of media and is limited but growing. Interactivity in narrative has been linked to increased transportation, increased identification or empathy, and increased engagement through the added pleasure of agency or control [43,44]. Even in text-based interactive narratives (which do not offer the seamless transitions of electronic media), decision points do not appear to interrupt the process of transportation [40]. The audience “enacts rather than witnesses the story,” and thereby “more deeply internalizes and personalizes the story events” [43]. As enjoyment is highly correlated with transportation, interactive narratives—by creating greater transportation—should be more enjoyable and lead to more sustainable change than traditional narrative forms [40].

Interactive narratives highlight the cause-and-effect relationships between the audience’s decisions and the consequences that ensue. In their largely theoretical examination of the psychological processes and outcomes of reader engagement with interactive narratives, Green and Jenkins [40] focus on increased player control and concomitant looser narrative structure as key characteristics. In addition to increased agency and control, Green and Jenkins posit that the audience’s response to interactive narrative is mediated through different roles of the self: as they make decisions for the characters, readers might reflect on similar situations in their own lives (“self-referencing”); they might explore possible selves and see how they feel and behave in different situations; and they might assume an increased sense of responsibility toward the characters. In their own empirical research, Green and Jenkins found that most people make decisions for a character based on what they personally would do in real life, by identifying with the character and putting themselves in his or her shoes, rather than by trying to understand a character’s unique motivations.

Some studies have explored the potential of the increased empathy fostered by this process of perspective-taking to reduce stigma toward marginalized groups [45,46]. A study of the effects of an interactive narrative on attitudes toward undocumented immigrants [47] suggests that simply adopting another person’s perspective in such a narrative may be an effective tool to help reduce prejudice toward marginalized social groups.

Some commentators frame interactivity in terms of experiential play that fosters curiosity, experimentation, and exploration, stressing entertainment and motivational dimensions [48]. A game that the audience can choose whether or not to play needs to be entertaining and *intrinsically motivating* [49], facilitating repeat exposure to the content. Repeat exposure is further facilitated by the potential narrative offers for parallel but superficially different storylines “that create interest, providing an opportunity for multiple exposures to critical material without making it seem redundant” [38]. This, in turn, helps to develop “gist” decision-making prior to or in the absence of conscious deliberation [50].

In our Theoretical Framework, these understandings of the opportunities afforded by interactive narrative translate into the following psychological process mediators: (1) *Identification;* (2) *Transportation/immersion;* (3) *Enjoyment/appreciation*; (4) *Sense of agency/control*; and (5) *Intrinsic motivation, supporting repeat exposure and attention/retention*.

### Social Behavioral Theory: Behavior Change Components and Behavioral Mediators

#### Social Cognitive Theory

Social Cognitive Theory (SCT) [51] is distinctive among social behavioral theories not only on account of its comprehensiveness but also for the fact that it provides operational insight to guide intervention design. As a mark of the relevance of SCT to games for health, Bandura has explicitly extended his theory not only to entertainment-education [52] but also to interactive media, including electronic games [53], citing the need “to make creative use of the revolutionary advances in interactive technologies” [54].

SCT provides a holistic account of the mediators and mechanisms of social behavior change communication that is grounded in an understanding of human agency as operating within a broad network of sociostructural influences. Bandura distinguishes between direct and indirect (ie, socially mediated) pathways of media influence: in the indirect pathway, media links people to social networks that can provide ongoing guidance, as well as natural incentives and social support, thereby linking the media world with the real world.

A central principle of SCT is that humans learn not only through direct personal trial-and-error experience but also through social *modeling* (also known as *observational learning*), that is to say, by observing others perform a behavior, either in our immediate environment or through the mass media. Models are a source of “inspiration, competencies, and motivation” [52] and can transmit knowledge, values, and skills, along with new behaviors and coping strategies. Models are most effective when they resemble the intended audience. Observing others achieve desired outcomes through their actions can create *outcome expectancies* that serve as motivators (or in the case of failures, as disincentives) or *reinforcement*. In the case of interactive role-playing narratives, observation is combined with active problem-solving and decision-making. *R* *einforcement* can also include the personal satisfaction or the social approval or disapproval that the behavior elicits (subjective norms) or, in the case of a game, the rewards system.

To enact behaviors, we need information and skills, but also a belief in our ability to use those skills, namely *self-efficacy*, the central mediator of behavioral change within SCT. The most effective way to instill self-efficacy is by combining modeling with *guided* opportunities to incrementally achieve *mastery*. Learning needs to be broken down into small manageable steps so that the participant gradually builds confidence. Through *repeat exposure* and practice with increasing challenges, we ideally learn incrementally how to manage setbacks and overcome obstacles with perseverance and effective strategies, without ever becoming demoralized.

Efficacy beliefs play an important role in shaping the course of people’s lives by influencing their outcome expectations and *goal-setting* [55] and, therefore, the types of activities and environments they choose. *Future orientation* associated with goal-setting gives one’s life direction, coherence, and meaning. We need both long-term and short-term goals to motivate and guide our behavior: goals have little impact unless they are translated into achievable subgoals and concrete plans and strategies for achieving them.

Because no 2 situations are alike, skills need to be adapted to suit varying circumstances. The necessary translation of observed learning to abstract skill is “greatly facilitated by having models verbalize their thoughts aloud as they engage in problem-solving activities,” making the thoughts guiding their decisions and action available for adoption [56]. Bandura has commented that health education for youth has focused on providing facts and has not equipped children with the skills and efficacy beliefs to manage social and emotional pressures to adopt behaviors that are detrimental to their health and manage social relationships. Integrating guided mastery with family and community efforts to provide social support is ideal [53]. Mobile health (mHealth) interventions have tended to focus on stand-alone interventions at the individual level [57]. Commentators have cautioned against mHealth efforts making a virtue of exploiting the affordances of mobile technologies to offer stand-alone interventions and “to practice public health at increasing distances” from communities [58]. Particularly in the case of games for preadolescent populations, they must seek to promote *p* *arent-child dialogue and social support*. This, in turn, can help reinforce *links between the virtual and real world*.

In our Theoretical Framework, these understandings translate into the following behavior change components: (1) *Modeling/Observational learning*; (2) *Problem-solving, decision-making, and related outcome expectancies*; (3) *Reinforcement (social, personal, and rewards)*; (4) *Guided mastery through cognitive and behavioral rehearsal in role-playing and skills-building exercises with feedback and virtual mentors*; (5) *Goal-setting (self and characters)*; and (6) *Links between virtual and real world*.

In addition, existing evidence-based HIV prevention interventions for youth provide the following behavioral mediators, drawing on theories including SCT, the Theory of Planned Behavior [59], and the Health Belief Model [60]: (1) *Knowledge*; (2) *Attitudes (behaviors, gender, relationships, and stigma)*; (3) *Risk assessment/perception*; (4) *Perceived social norms*; (5) *Future orientation*; (6) *Behavioral intentions*; (7) *Behavioral skills and related self-efficacy*; and (8) *Parent-child dialogue and social support*.

#### Constructivist Learning Theory and Curriculum Development

Drawing on a constructivist model [61], our Instructional Components overlap with SCT. They focus on *active learning* in a dynamic, but structured, learning environment designed to foster student meaning-making and problem-solving around specific *scaffolded learning objectives*. Storylines grounded in research on young Africans’ narratives are designed to create *relevance* and *value*, while learning is distributed and reinforced across a range of *multimodal* (drawing on a range of sensory modalities) and complementary game components.

## Game Structure

In the following section, we present the structure of *Tumaini*, which is made up of 3 parts: (1) an interactive role-playing narrative; (2) a set of mini-games; and (3) “My Story,” in which the player reflects on how the game relates to his/her life.

The interactive role-playing narrative is about 3 boys and 3 girls who are aged between 11 and 14 years at the start of the game. Players role-play each of the 6 characters (male and female) as they age over the course of the 3 game levels, passing into or through adolescence. The characters face real-life challenges that the players are likely to face at some stage in their own lives. These include peer pressure; puberty; violence; and decisions about smoking, alcohol, drugs, and sex. The player makes choices for these characters and sees the consequences of those choices in the characters’ lives. The game is made up of 18 chapters distributed over 3 levels. The outcomes of the players’ decisions find expression in over 40 different potential epilogues across the 6 characters. Major decision points are designed to be meaningful and to drive the story (if incrementally so), increasing players’ sense of agency.

The entire game comprises approximately 12 hours of discrete gameplay. It is designed to be replayed so that players can observe the outcomes of different decisions, exploring possible selves through the different characters and their various paths through the game. Replay is encouraged via the rewards system, such that additional prizes are awarded for completion of new decision paths. Cliffhangers at the end of each level are designed to stimulate curiosity, elicit anticipation of possible outcomes, and increase enjoyment/appreciation, thereby increasing intrinsic motivation.

The player is positioned immersively as a third-person character who is addressed directly by the playable characters (eg, “Do you ever feel unsure of yourself?”) and has access to their thoughts. In addition to deciding the characters’ actions from a brief menu of options in response to “What should I do/say?” prompts, the player is also invited to identify characters’ feelings in response to “How do I feel?” prompts, fostering increased identification with characters, self-referencing, and emotional rehearsal. Each character has an older mentor, modeling adult-child dialogue, and social support. The mentor distills out attitudinal and behavioral learning from the experiences of the characters (eg, around gender, relationships, and stigma). Each chapter is accompanied by either a mini-game or a *My Story* component, and a prize is awarded for completion of each chapter or *My Story* component and for successful completion (a score of at least 80%) of a mini-game.

The mini-games are designed to build on the interactive narrative, reinforcing knowledge, skills, and related self-efficacy through guided mastery (cognitive and behavioral rehearsal through role-playing and skills-building exercises with feedback and virtual social support). Some of the mini-games are quizzes; some ask players to assess the risk of specific situations; some ask the player to respond to pressure situations; and some are jigsaws. The topics of the mini-games are connected to the topics in the role-playing story. For example, when the main story is about puberty, the players play the puberty game. When the character in the main story is being pressured by his friends to drink alcohol, players play a skills-based game about saying no to peer pressure.

In the mini-games, the player is challenged with questions of increasing difficulty and receives feedback, encouragement, and suggestions. Right answers are applauded, whereas wrong answers elicit a deflated sound, followed by feedback from 1 of the characters (eg, “You could have said...”). Reinforcement takes multiple forms: vicarious internal reinforcement is modeled through the playable characters and their outcomes, vicarious social reinforcement is modeled through the reactions of peers and mentors, and the players’ learning is reinforced through growing personal mastery and in-game rewards.

The third part of the game is called “My Story.” This component of the game asks players to think about different aspects of their personalities and to set personalized goals. It allows them to connect the knowledge and skills they learn in the game with their own lives. Like the mini-games, the topics for this part of the game are connected to the main role-playing story. For example, when the characters are considering what future goals they have, players are asked to identify their goals and what they can do now to help themselves reach them, reinforcing behavioral intentions. At the end of each chapter, a “bridge” question (“What about you?”) links the chapter’s narrative to the player’s real world, encouraging cognitive rehearsal and connecting knowledge and skills to a personal decision-making framework infused with future orientation.

## Discussion

### Overview

We have shared the multidisciplinary grounding informing the design and the process and outcome mediators of *Tumaini,* which draws on insights from fields including communication, education, psychology, anthropology, educational gaming, and public health. Making explicit the theoretical grounding of a game-based intervention allows us to articulate our assumptions and strategies, anticipate outcomes and evaluate impacts (including intermediate effects), and thereby understand pathways to change, with a view to ultimately contributing to the development of more effective games. It also helps us to strengthen the credibility and improve the accountability of games for health and to help move the field forward. We now situate *Tumaini* in the context of comparable narrative or game-based interventions and within discussion of the theory and practice of using interactive narrative in health promotion, with a view to furthering theoretical elaboration.

### The Role of Narrative in Games

The question of what role narrative should play in games for health has elicited divergent opinions [62]. Stories have played central or peripheral roles in serious games, often depending on the game’s behavioral objectives and, concomitantly, its pedagogical approach. Narrative has tended to serve a secondary and, above all, motivational role in games for chronic disease prevention, where behavioral decision-making is more deeply rooted in habitual day-to-day living; hence, story and situation may occupy a less central place. Its role in a sexual health game is, in contrast, likely to be much more central, particularly if the game is for young adolescents who are learning to read and navigate situations of potential sexual risk for the first time: situation-based cognitive and behavioral rehearsal is essential to help them prepare. It is nonetheless notable that the structure of our Theoretical Framework, integrating intervention components, mediators, and outcomes, draws inspiration from that of Thompson et al [29] for a narrative-based game designed to prevent Type 2 diabetes and obesity among young people. In short, narrative has relevance whenever we are seeking to facilitate the transfer of behavioral skills to real life, whether it is choosing where to set limits on a first date or choosing wisely when ordering a meal at a restaurant.

There was for a time a contentious debate within the game literature about narrative-oriented versus game-oriented approaches (the “narratology vs ludology debate”). By way of conciliation, Ryan [63] differentiates between the *narrative game*, in which story is secondary to gameplay, and the *playable story*, in which gameplay is secondary to story. She relates this distinction to 2 forms of play: *ludus* (more rule-bound, competitive, and focused on problem-solving) and *paidia* (imaginative play, eg, make-believe). Unlike *ludus* games, *paidia* games do not lead to winning or losing. Driven by stories based on decision trees, *Tumaini* ’s central structure is more aligned with *paidia* and the *playable story* than with *ludus* and the *narrative game*. It cannot be won or lost, and no strategic game-based thinking is involved.

*PlayForward: Elm City Stories,* a game for a similarly aged US minority population, has comparable HIV education and risk reduction goals. In contrast to *Tumaini*, however, *PlayForward* is a winnable game and would be situated more toward the *ludus* /*narrative game* end of Ryan’s spectrum. In *PlayForward*, a set of mini-games are the primary vehicles for delivery of intervention content related to knowledge and skills. Each mini-game focuses on a specific set of behavioral or motivational outcomes. After successfully engaging with a series of stories representing risk situations teens face in middle and high school, players acquire “senses” and “powers” by playing the skills-based mini-games [64]. The People Sense mini-game, for example, is intended to increase adolescents’ “knowledge about social dynamics, risk taking, and the interaction of social dynamics and risk taking.” Here, players learn to navigate peer relationships by identifying peers who may be more likely to engage in risky behaviors. They manipulate the physical closeness of individuals to their own avatar to graphically represent a distancing of risk. *PlayForward* has been shown in RCT to improve knowledge and attitudes [65].

In contrast to *PlayForward* ’s more cognitive model, *Tumaini* seeks to foster experiential and intuitive contextualized learning through its interactive narrative-based approach. The player observes peer pressure in action, makes decisions for the characters facing it, and follows the consequences. Older non-playable characters act as mentors and distill key messages from these experiences. The player is asked whether she/he has faced similar situations in real life (“What about you?”) and to reflect on how they might react in these situations. The player then has an opportunity to practice skills in responding to peer pressure scenarios in a mini-game, in which she/he must select a response and is given feedback by 1 of the game characters acting as a virtual mentor. It is hoped that this will allow players to intuitively extract general rules and situational skills from the specific scenarios they are exposed to in the role-playing game (through multiple parallel but nonredundant examples) and apply them in their own lives. Repeat exposure to parallel situations should help build an experience-based, emotionally charged, and intuitive “gist” learning [50].

### Interactive Narrative in Sexual Health Games

Sexual health interventions rooted in interactive narrative have been referred to by various names, depending on the extent of their narrative content and their medium (and hence technological affordances). In his 2011 review of computer technology-based interventions in HIV prevention, Noar identified “virtual decision-making interventions” as “interventions that simulate dating and sexual situations and allow the user to make choices at various decision points and witness the consequences of various (good and/or bad) decisions” [66]. In 2015, Muessig et al termed “Virtual Reality scenarios” those that are designed to “increase mastery of important skills...address affect management, and provide more robust situation-specific intervention techniques (eg, practicing HIV status disclosure or condom negotiation skills)” [4]. Key examples of such interventions for our purposes are *Socially Optimized Learning in Virtual Environments* *(SOLVE)* [67] and *What Could You Do?* (subsequently updated and expanded into the interactive video-based intervention *Seventeen Days*) [68]. Both interventions were pioneering interactive interventions in the years before new digital media and have evolved with technological advances. Both interventions feature “virtual dates” in which the user makes choices for the characters that lead them toward or away from unsafe sex. However, where *SOLVE* is for men who have sex with men (MSM) and is now delivered via the Web, having been rebuilt for a game platform, *Seventeen Days* (and its precursor) is an interactive video for heterosexually active female teenagers and is designed for delivery in health clinics.

*SOLVE* invites MSM to participate in an interactive virtual environment designed to “simulate the emotional, interpersonal, and contextual narrative of an actual sexual encounter while challenging and changing MSM’s more automatic patterns of risky responses” [69]. As participants role-play characters in socially engaging and emotionally realistic sexual situations, their learning is scaffolded by supportive, and often humorous, virtual peer counselors who provide advice and possible negotiation tactics. *SOLVE* counselors can “make clients’ emotions, cognitions, goals, problem solving, and decision-making steps more salient, especially when these are leading to risky outcomes.” Those who received the interactive video in addition to counseling reduced risky sex and increased protected sex compared with those who received counseling alone [69].

Although intended for a different audience, *Tumaini* bears some similarities to *SOLVE*, not least by virtue of its focus on *affect*. The player is asked not only to make choices at decision points faced by the virtual characters but also to identify with their emotional state through the “inner voice” (“How do I feel?”). It uses virtual mentors (sometimes referred to as “pedagogical agents”) in the form of non-playable adult and peer characters to provide guidance in the role-playing game and to provide feedback on exercises in the mini-games. Where *SOLVE* focuses on self-regulation for sexual decision-making among MSM, our focus on a preadolescent, prerisk population meant that we placed greater emphasis on cognitive and behavioral rehearsal for situations our players had not yet encountered and related outcome expectancies. We are currently developing an iteration for older adolescents, in which this balance is likely to shift.

In addition to interactive vignettes, *Seventeen Days* includes a condom demonstration scene and “mini-documentaries.” Based on formative research, the intervention was designed to address women’s perceived lack of control over sexual situations. It sought to show teenage girls that sex is not something that just happens and that they have choices in sexually charged situations. In the initial RCT, the interactive *What Could You Do?* video increased reported abstinence and reduced reported condom failures and sexually transmitted infection diagnoses post intervention compared with 2 comparison groups [68]. In the subsequent RCT, participants in the *Seventeen Days* arm reported higher perceived condom acquisition self-efficacy after 6 months [70].

Built on the science of decision-making [3], *Seventeen Days*, like *Tumaini*, seeks to help adolescents develop proficiency in identifying and evaluating risk situations before their exposure to them. It is distinctive for the emphasis it places on cognitive rehearsal. At various points, the screen freezes for 30 seconds and the user is invited to mentally rehearse what she would do in this situation, for example, “What could you do if you didn’t want to go off alone with a guy? Think about it, and practice it in your head.” Downs et al [3] summarize their interactive narrative-based strategy:

We sought to create a mental model that afforded the active mastery needed to absorb future information and experiences, and to make inferences about unfamiliar situations...By helping viewers to identify choice points, analyze their impacts, and rehearse potential responses in advance, the intervention sought to empower young women to create their own, alternative, practiced scripts.

As a prerisk prevention intervention for preadolescents, *Tumaini* as currently designed for a general 11- to 14-year-old audience does not explicitly invite cognitive rehearsal in sexually charged situations. However, it consistently seeks to link the virtual world with the real world. For example, in “What about you?” questions at the end of each chapter, the player is invited to relate events in the role-playing narratives, such as when a character is pressured by an older adult to have sex, to their own lives (“Who would you talk to if someone older than you tried to put pressure on you to have sex?”). Similarly, the *My Story* reflection and goal-setting components are coordinated with the content of the role-playing game such that during a chapter when a character wrestles with norms of masculinity, the player is invited to select values he/she has about what kind of man/woman he/she wants to be. More research is needed to better understand players’ thought processes when making decisions in the role-playing game.

The player is not only invited to decide the actions of the character but also to choose between potential emotional states. For example, when Ruth finds out that a boy likes her, the player must decide whether she is feeling *Flattered*, *Nervous*, or *Excited* about the prospect. We believe this helps the player put him or herself in the shoes of the character and become emotionally immersed, leading to cognitive rehearsal on a direct, personal level. More research is needed to understand if the tendency observed in Green and Jenkins’s research whereby readers of interactive narrative make the choices they would make in real life carries over to our preadolescent Kenyan participants.

*Tumaini* differs from *Seventeen Days* and from *SOLVE* by virtue of the extent and thematic scope of its interactive narrative format. Where the other interventions are based on vignettes, *Tumaini* has a full narrative structure. Each of *Tumaini* ’s 18 chapters is devoted to 1 of the 6 characters. Although the characters represent a range of socioeconomic backgrounds, they all move within the same geographic universe, represented by a town with primary and secondary schools, restaurant, clinic, etc. In short, although *Tumaini* ’s interactivity aligns with *SOLVE* or *Seventeen Days*, its narrative structure is more akin to an entertainment-education soap opera [71].

*PlayForward*, *SOLVE,* and *Seventeen Days* were designed for US populations who have greater ease of access to interactive digital technologies than the population for which *Tumaini* was designed. There are, however, precedents for the use of interactive narrative for HIV prevention in sub-Saharan Africa. *Three and a Half Lives of Philip Wetu* is a 30-min interactive HIV prevention video made in Namibia that allows the audience to make choices for the male protagonist who has multiple concurrent female partners [72]. The interactive video is distinctive for being designed for facilitated use in a group setting. At the 3 decision points in the story, the audience is invited to discuss what has happened so far and decide what Philip Wetu should do next (eg, whether he should get tested). The film then continues in line with the audience’s decision. *SwaziYolo*, in contrast, is an interactive smartphone game designed for individual use by young adults aged 18-29 years in Swaziland. In *SwaziYolo*, the player assumes the role of a young adult looking for love in Swaziland, making decisions about relationships and sexual health. The game consists of 2 parts—an imaginary social network and a series of dates in nightclubs and cafes. The player’s choices between different potential courses of action affect his or her own health and safety and the opinions and behaviors of other characters; thus, the goal is to stay healthy and happy while maintaining relationships with other characters. *SwaziYolo* is currently being trialed in an RCT [73].

### Theoretical Elaboration and Areas for Further Research

Within the humanities-focused literature, interactive digital narrative (IDN) has been described as extending “from avant-garde art to interactive documentaries, electronic literature, video game design, applications of artificial intelligence (AI) research and ubiquitous computing” [41]. Buoyed by the horizons opened by AI, scholars have suggested that IDN “bestows cocreative power on its users” and “promises to dissolve the division between active creator and passive audience and herald the advent of a new triadic relationship between creator, dynamic narrative artefact and audience-turned- participant” [41]. As technological possibilities have evolved, public health applications of interactive narrative have grown alongside artistic experimentation and the deployment of increasingly complex algorithmic possibilities.

With hundreds of decision points—many with consequences for the plot, a few, without—across its 18 chapters, *Tumaini* offers an extensive array of possible “instantiated narratives” [74]—and over 40 possible outcomes (represented by discrete epilogues) across the 6 characters. These narrative permutations, although extensive, are certainly not limitless as is envisaged by some AI-driven interactive narratives. Within applied uses of interactive narrative, the choices offered to players are likely to remain circumscribed in line with the game’s learning objectives. It is nonetheless of value for public health to follow the evolving theory and practice of interactive narrative in other disciplines.

Given the importance of interactive narrative for skills-building through cognitive and behavioral rehearsal, development of outcome expectancies, and modeling, there is considerable need for further research in the field. Like the interventions described above, research on interactive narrative has covered a range of media and platforms, from written texts [40], through interactive television [43], to our own smartphone game. Although there is potential for cross-medium learning, there is also a need to differentiate between the respective effects of different platforms. Pressing areas for further research also include the need to better understand (1) players’ mental processes while navigating and making decisions within an interactive narrative, including their motivations for choosing one path over another; (2) whether the perspective of the player (first vs third person) affects transportation and identification; and (3) the differential impact of different paths through the narrative and accompanying loss or gain frames [40].

Although Voderer et al [75] have suggested that interactive narratives are only beneficial to audiences with higher cognitive capacities, the quantitative and qualitative findings from our pilot study of *Tumaini* with very young adolescents (and the longstanding youth appeal of CYOA novels) suggest otherwise. In our pilot study, *Tumaini* functioned as a stand-alone intervention for preadolescents. However, we plan to bolster parent-child communication, social support, and linkage to services within a more extensive intervention package addressing a wider range of audiences within a socio-ecological framework.

Our Theoretical Framework separates psychological process mediators, drawn from communication theory, from behavioral outcome mediators, drawn from social behavioral theory. However, there is evidence of direct linkage between, for example, transportation and attitudes. In our 2017 pilot of *Tumaini* in Western Kenya, we did not measure the psychological process mediators identified in our Theoretical Framework because of concerns around response burden for our preadolescent participants. Various scales assessing these mediators are available for adaptation to our audience. Mediation analysis, facilitated by log files generated by our game, will allow us to better parse out linkages of these cross-disciplinary mediators and better understand the mechanisms of effect of our intervention. This will, in turn, increase understanding of processes of health behavior change and support the development of increasingly effective games for health interventions.

### Conclusions

Calls for improved behavioral theories for mHealth interventions have arisen from the need to better describe process and effects of interactive, adaptive, and dynamic interventions at the intraindividual [14] and social levels [16]. Although it is unlikely that we will dispense with well-established behavioral mediators, there is an increasing need to build new theoretical frameworks drawing on literature informed by disciplines ranging from computer science through the cognitive and behavioral sciences to the social sciences and humanities.

The increasing availability of smartphones, including in low-income countries, offers unprecedented opportunities to take advantage of the potential for interactive narrative to contribute to health promotion efforts. We have shared the multidisciplinary theoretical framework that guides *Tumaini*, a smartphone-based HIV prevention game. The development and sharing of multidisciplinary theoretical frameworks, combined with studies incorporating multilevel mediation analyses, could contribute to the development of new theoretical frameworks to guide and explain the effects of games for health and other mHealth interventions and thereby increase their effectiveness.

## Appendix

Multimedia Appendix 1 Sample graphics from Tumaini.

## Abbreviations

AI: artificial intelligence
CYOA: choose-your-own-adventure
IDN: interactive digital narrative
mHealth: mobile health
MSM: men who have sex with men
RCT: randomized controlled trial
SCT: Social Cognitive Theory
SOLVE: Socially Optimized Learning in Virtual Environments
