# Supplementary figures and images for: Interactive Narrative in a Mobile Health Behavioral Intervention (Tumaini): Theoretical Grounding and Structure of a Smartphone Game to Prevent HIV Among Young Africans
Source: JMIR Serious Games. 2019 May 8;7(2):e13037. doi: 10.2196/13037 (PMC6530263; doi:10.2196/13037)

**Sample graphic from narrative:**

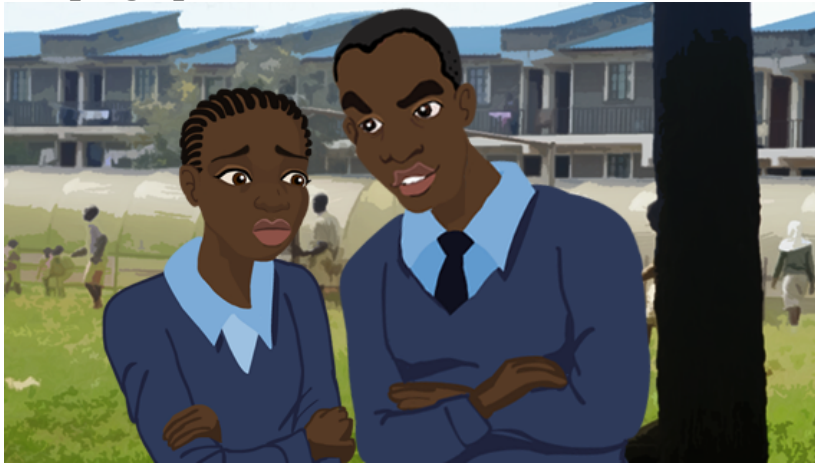

**Sample graphic from mini-games:**

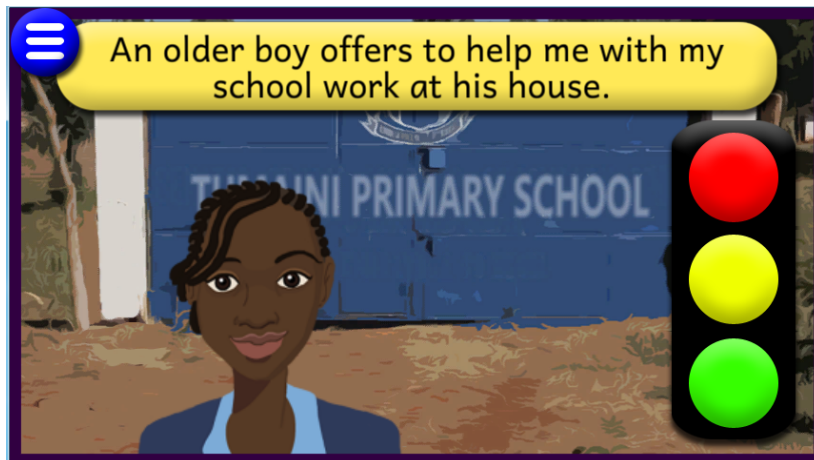

**Sample graphic from My Story:**

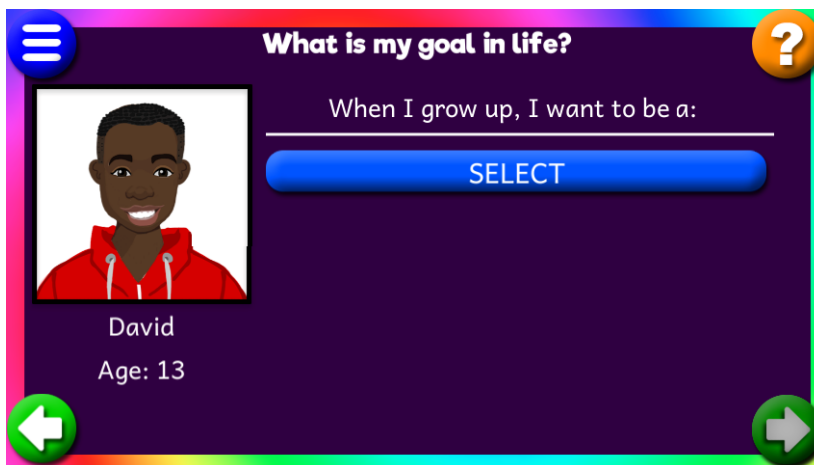

Supplement: Multimedia Appendix 1 [file games_v7i2e13037_app1.pdf]
